# A Usage-Based Proposal for Argument Structure of Directional Verbs in American Sign Language

**DOI:** 10.3389/fpsyg.2022.808493

**Published:** 2022-05-17

**Authors:** Lynn Hou

**Affiliations:** Department of Linguistics, University of California, Santa Barbara, Santa Barbara, CA, United States

**Keywords:** verb agreement, argument structure, usage-based linguistics, construction grammar, American Sign Language

## Abstract

Verb agreement in signed languages has received substantial attention for a long time. Despite the numerous analyses about the linguistic status of verb agreement, there is little discussion about the argument structure associated with “directional verbs,” also known as agreeing/agreement or indicating verbs. This paper proposes a usage-based approach for analyzing argument structure constructions of directional verbs in American Sign Language (ASL). The proposal offers low-level constructions for reported speech, non-dedicated passive and reflexive, and stance verb constructions, which capture the patterns, abstracted from recurring usage events, that are part of users’ linguistic knowledge. The approach has potential to push the field of sign linguistics in new directions of understanding the interplay of language use and structure.

## Introduction

**FIGURE 1:**
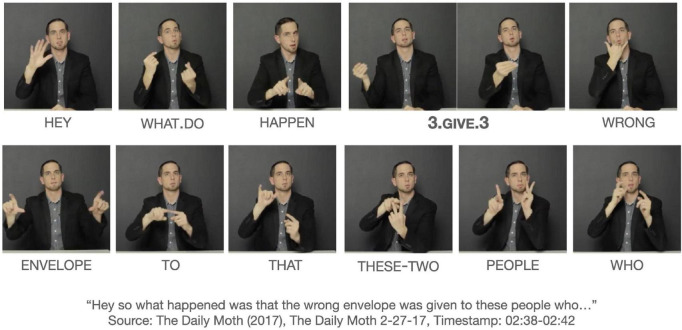
The use of GIVE in an argument structure construction. Images produced with permission, source information available in the Supplementary Material.

We use verbs to discuss events and situations in everyday life. Verbs are the canonical predicates and generally express the action in a clause. This is true for any human language. What distinguishes signed languages from spoken languages is the use of space. Signers capitalize on the signing space to produce verbs, which often can appear to be an iconic or transparent conceptualization of events. Some verbs express the transfer of an object in the signing space, or the surrounding physical space that encompasses the signer’s body. Such verbs are commonly labeled as *agreeing* (or *agreement*), *directional*, or *indicating* verbs. The terminological choice depends on the researcher’s theoretical stance. Figure 1 shows the production of the beginning of a complex clause in American Sign Language (ASL) in which the verb GIVE is represented as a sequence of initial and final locations. The initial location of the verb starts on the signer’s right and the final location ends ahead of the signer. From looking at the verb form alone, one could get a prototypical interpretation of an agent/subject giving a theme to a recipient, i.e., “s/he/they gave it to you/her/him/them” and may be considered an instance of “verb agreement.” Yet the larger construction in which the verb form occurs gives a rather different interpretation. An object is given to the recipient, who is identified as two people, while the agent is unspecified, as there is no antecedent or an overly expressed referent to identify it (Janzen et al., 2001; Leeson, 2001; Rankin, 2013; Barberà and Cabredo Hofherr, 2017; Nordlund, 2019). The construction functions as an agent defocusing construction, which may be either a passive construction or a R(eference)-impersonal construction, depending on one’s view. This example suggest that the argument structure does not entirely come from the semantics of the verb, i.e., the lexicon but rather the meaning of the argument structure construction contributes to the verb in it too (Goldberg, 1995, 2006; Diessel, 2015).

I take the above example as a starting point to discuss the scholarly bias toward “verb agreement” in signed languages. There are various morphological and syntactic analyses of verb agreement in signed languages (Padden, 1988; Meir, 2002; Mathur and Rathmann, 2010, 2012; Pfau et al., 2018; Quer, 2021, a.o.). There is also an extensive debate over the linguistic status of verb agreement (Liddell, 2003; Lillo-Martin and Meier, 2011; Fenlon et al., 2018; Schembri et al., 2018, a.o.). Some existing analyses rely on contextually isolated, elicited data, as the focus of these analyses is more theoretically abstract than empirical. The emphasis has been on positing linguistic mechanisms, both modality-independent and modality-specific, for the instantiation of agreement. Other analyses have used large-scale datasets such as corpora to test empirical observations about linguistic and social factors that may predict the spatial modification of verbs (de Beuzeville et al., 2009; Fenlon et al., 2018). Those analyses, regardless of the theoretical and methodological differences, are similar in their concentration of the morphosyntactic and syntactic properties of the verbs and the instantiation of agreement (or the lack thereof).

By comparison there has been little discussion of the argument structure constructions of directional verbs. The few exceptions are argument structure alternations of different verb classes from generative, cognitive, and typological perspectives (Kegl, 1990; Kimmelman, 2018, 2022; Johnston, 2019b; Oomen, 2020). However, there is little discussion argument structure constructions from a *usage-based* perspective with the exception of Johnston (2019b) who focuses on Australian Sign Language (Auslan). Usage-based linguistics tend to look at the occurrence of directional (and non-directional) verbs, the presence and absence of core arguments, and the realization of grammatical relations in a diverse range of argument structure constructions, ideally from a corpus (Johnston, 2019b), and posit abstract generalizations based on these constructions. Figure 1 is such an example. This raises an empirical question: what argument structure constructions do directional verbs participate in and what kind of generalizations can be abstracted from these constructions? Directional verbs make an intriguing case study – they are transitive and can mark the core arguments in the signing space, though not all transitive verbs are directional verbs.

This is a position paper that argues for how a usage-based approach can advance our understanding of directional verbs in argument structure constructions in ASL (and perhaps by extension, many other signed languages). The goal is not to present a basic description of argument structure of all directional verbs *per se*, but rather to spotlight a few types of low-level constructions, or templates, in which directional verbs occur and to discuss how these constructions can expand our understanding of verbs and more broadly, ASL grammar. The constructions of interest are reported speech, non-dedicated passive/reflexive, and stance verb constructions, all which involve directional verbs. There is growing research on active and passive constructions that occur with different verbs (Janzen et al., 2001; Leeson, 2001; Rankin, 2013; Barberà and Cabredo Hofherr, 2017; Nordlund, 2019) and also recent research on a family of stance verbs (Hou, 2022). I build on the previous research with additional data from the internet and present a preliminary usage-based analysis that includes low-level constructions for argument structure of directional verbs.

The paper is organized as follows. First, there is a brief introduction to usage-based linguistics with a focus on argument structure constructions. Next, there is an overview of verb agreement in signed languages; the overview covers various theoretical perspectives. Then there is a discussion of data and methods, followed by a preliminary analysis of verb constructions. Finally, the paper wraps with some suggested directions for advancing research on argument structure constructions.

### Usage-Based Linguistics in a Nutshell

The analysis is based on a few fundamental assumptions about usage-based linguistics. First, language structure emerges from language use, and various aspects of the structure is constantly reshaped by continued use (Barlow and Kemmer, 1994; Bybee, 2010; Christiansen and Chater, 2018; Diessel, 2019). Language use is viewed as a dynamic product of domain-general cognitive processes, not based on a “language module” that contains rules for generating sentences. Users – speakers and signers – develop an abstract representation of grammatical knowledge from their experience of (re)using words in utterances (Langacker, 1987; Fillmore et al., 1988; Croft and Cruse, 2004; Bybee, 2006; Goldberg, 2006; Elman, 2009; Dąbrowska, 2014; Lepic and Occhino, 2018; Wilkinson et al., in press). The lexicon and grammar are not treated as separate components of linguistic knowledge with linking rules. Rather, linguistic knowledge is represented as a hierarchical network of constructions, learned pairings of form with semantic of discourse functions, that are organized and connected by taxonomic links (Croft and Cruse, 2004; Goldberg, 2006; Langacker, 2008). The constructions come in all sizes, ranging from words to complex constructions and varying along the dimensions of schematicity and specificity. Under this view, Example (1) contains twelve listed constructions that encompass individual words and syntactic clauses. A user can combine different constructions to produce an actual expression, provided that the constructions do not conflict with one another in according to one’s grammar.


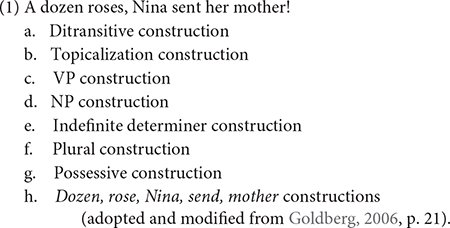


Second, grammar is viewed as the cognitive organization of one’s experience with language (Bybee, 2006, 2010). A user abstracts all linguistic expressions from recurrent usage events, or actual language uses, and categorize them based on similarities. Frequency plays a prominent role in the experience. The more frequent expressions with specialized functions are more entrenched, or committed to and stored in long-term memory; each additional instance of an expression strengthens its representation (Bybee, 2006). The categories are not static, but can shift and change over time, as the user accumulates more experience in the world. In the case of argument structure constructions, a ditransitive construction [*subject verb object_1_ object_2_*] in English language varieties would be entrenched in the user’s grammar. This construction has a very high frequency of instances with different verbs such as *send, give, tell*, etc. that can occupy the verb slot. Other constructions vary in degrees of schematicity, or productivity, depending on one’s idiolect. According to Goldberg (1995), Croft and Cruse (2004), the comparison of the Caused Motion construction [*subject verb object_2_ to object_1_*] and the Ditransitive construction [*subject verb object_1_ object_2_*] reveals a difference in the patterning of semantically similar verbs, *tell* and *whisper.* Examples (2) and (4) show that *tell* and *whisper* participate in the Caused Motion construction, but only *tell* participates in the Ditransitive construction. This suggests that some verbs can overlap with both constructions, but other verbs cannot. One construction is more schematic than the other construction.


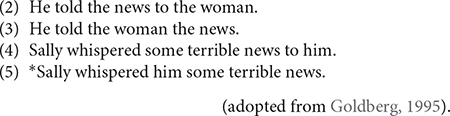


Finally, the term “argument structure constructions” implies a theoretical stance about the lexical representation of verbs. The meaning of the verb alone does not always determine the realization of the argument structure, i.e., the core arguments of the event and the syntactic expression corresponding to a specific meaning. Rather it is the syntax, or the whole syntactic construction, a learned pairing of form and meaning, that contributes to argument realization (Goldberg, 1995, 2006). Moreover, verbs are associated with specific argument structure constructions that repeatedly occur in language use. These constructions are organized in a hierarchical network in which constructions are represented at different levels of schematicity and connected by taxonomic links.

To the best of my knowledge, there has been very little research on argument structure constructions in signed languages. One major reason for the gap may be the ongoing controversy over the linguistic status of verb agreement morphology, which is discussed next.

## Background on Verb Agreement in Signed Languages

The interest in verbs has long occupied the academic field of sign language linguistics, which was heralded by the pioneering publications of Stokoe (1960), Stokoe et al. (1965), *The Dictionary of American Sign Language*. Early on, verbs received substantial attention for their interaction with the signing space^1^. Many researchers documented their observations about such verbs in ASL. Friedman (1975) called them *multidirectional verbs*, listing only six verbs as examples: “give,” “bring/take,” “borrow/lend,” “tell,” “go/come,” and “see.” The verbs and their arguments are analyzed from a thematic role perspective: arguments are marked for their thematic roles by means of directionality, or “direction of action from source to goal” (955). The arguments’ locations determine the verb’s direction of path movement and palm orientation. Friedman stated that ASL lacked true pronouns, but pointing encoded pronominal reference for first person, second person, and third person, and the directionality of the verbs did likewise. A change in the direction of path movement and orientation signals a change in the meaning of the sentence in which the verb occurs, hence the name *multidirectional.* As Friedman was giving a general description of the expression of space, time, and person reference in ASL, the discussion of multidirectional verbs was superficial.

Fischer and Gough (1978) investigated the variety of morphosyntactic properties of a bigger sample of ASL verbs that were taken from Stokoe et al. (1965). Like Friedman, Fischer and Gough observed that some verbs exhibit changes in the direction of path movement and/or palm orientation to mark the arguments and labeled these verbs as *directional verbs.* This term is sometimes still used to this day. It was also observed that some verbs can be reversible by changing the direction of the path movement and/or the facing of palm orientation. Other verbs are phonologically constrained from exhibiting path movement, but signers can produce them in various spatial locations. Such “locatable” verbs point to their arguments through the facing of palm orientation. A sign OWE was listed as such an example; one can mark the object of this sign by producing it near a spatial location associated with an argument. In Fischer and Gough’s view, ASL did have pronouns to mark person, and pronominal arguments were incorporated in the verbs. The processes of directionality, reversibility, and locatability enabled verbs to mark grammatical relations such as the subject and the object.

Padden (1988) took the analysis of directionality to a new level by classifying ASL verbs as a tripartite system of verbs: *plain*, *spatial*, and *inflecting*. This work builds on the aforementioned earlier works as well as Klima and Bellugi (1979), Meier (1982), Supalla (1982), and various unpublished talks and manuscripts. In Padden’s account, verbs are distinguished by their ability to participate in agreement. Central to marking agreement is the type of arguments, not just the directionality, reversibility, or locatability processes. Spatial verbs such as MOVE and PUT “agree” with locative arguments. Plain verbs cannot agree with their arguments; instead, they indicate grammatical relations by means of word order. Inflecting verbs “agree” with animate arguments for person and number. The term *inflecting* was later replaced with *agreeing* or *agreement*, since it was shown that plain verbs can inflect for aspect. Agreeing verbs have incorporated subject and object agreement markers; the initial and final locations of the verb are interpreted to mark core arguments, particularly the object.

### Theoretical Views of Verb Agreement

Many signed language researchers have adopted and adapted Padden’s analysis for describing and documenting verbs in ASL and many other signed languages. There is extensive research on this topic, as many researchers have detailed the formational properties of verb agreement. This research agenda is ongoing to this day; the agenda has been expanded and enriched by recent corpus and experimental studies. Much of the current literature suggests that the theoretical differences in analyzing the verbs would be grouped in two camps of linguistics, though this is becoming unmerited. The first camp is formal-generative linguistics. Researchers who are affiliated with this camp adopt some variation of the verb agreement analysis (Padden, 1988; Janis, 1995; Mathur, 2000; Meir, 2002; Rathmann and Mathur, 2002; Sandler and Lillo-Martin, 2006; Thompson et al., 2006; Mathur and Rathmann, 2010, 2012; Hosemann, 2011; Lillo-Martin and Meier, 2011; Wilbur, 2013; Costello, 2016; Pfau et al., 2018; Quer, 2021; a.o.). There appear to be several accounts, of which two are mentioned here (see Pfau et al., 2018; Schembri et al., 2018 for a recent review). Some researchers treat agreement as a morphosyntactic phenomenon, taking a syntax-semantics interface approach, while other researchers take a purely syntactic approach. What all these researchers have in common is that they tend to examine the formal structures independently of semantic or discourse functions; this is implied by how these approaches generally understand agreement as a syntactic relation between two linguistic elements. One element, the target, copies the morphosyntactic features of another element, the controller, so both elements encode the same features (Steele, 1978; Lehmann, 1982; Corbett, 2006). The corollary is that there are multiple various accounts that attempt to explain the phenomenon of agreement as a syntactic relation.

The second camp has been historically associated with cognitive linguistics, but the diversification of theoretical frameworks suggests that “cognitive linguistics” is too broad of an appropriate label for these frameworks. In the past, many researchers adopted the “indicating verbs” analysis in rejection of the verb agreement analysis (Liddell, 1995, 2000, 2003, 2011; de Beuzeville et al., 2009; Cormier et al., 2015a; Fenlon et al., 2018; Schembri et al., 2018). The indicating verbs analysis was originally inspired by the theory of mental spaces (Fauconnier, 1985) and are said to be “capable of being meaningfully directed to space toward entities, directions, or places” (Liddell, 2003, p. 97). By this definition, indicating verbs refer to both spatial and agreeing verbs. The directionality of the verbs is motivated by the actual or imagined locations of referents in the physical world. These verbs point to the referents, which are viewed analogous to pointing gestures by gesturers who employ similar cognitive mechanisms for referring to referents (see Fenlon et al., 2019 for a quantitative differences between pointing by signers and gesturers). Indicating verbs thus are viewed as a fusion of linguistic and gestural elements, or more specifically, “a structured composite construction of sign and co-sign gesture, similar to multimodal constructions of speech and co-speech gesture” (Schembri et al., 2018, p. 13). In an indicating verb, the handshape, orientation, and movement are morphemic and lexically specified, whereas the initial and final locations of the verb are not morphemic, i.e., gestural, and variable. The locations are not listable and predictable, since there may be an infinite number of possible locations, so a more plausible explanation is that they are motivated by the physical world or by the signer’s cognitive representation. This view treats verb agreement what I call a *morphemic-gesture* relation.

Some researchers who adopt the morphemic-gesture relation do not research indicating verbs exclusively but rather take a “neo-Peircean” semiotic approach to linguistic analysis of signed languages more broadly. They are more interested in investigating the diversity of semiotic resources for marking reference – and more broadly, for producing composite utterances (Ferrara and Hodge, 2018; Hodge et al., 2019; Johnston, 2019b; Puupponen, 2019). The indicating verbs are merely one of the many semiotic resources that deaf signers exploit for marking referents as part of composite utterances in spontaneous discourse. Other researchers take a “semiological” approach based on the “enunciation theories” for similar purposes with a different categorization of various signs and a greater emphasis on the functions of non-manual elements especially eye gaze (Cuxac, 1999; Fusellier-Souza, 2006; Cuxac and Sallandre, 2007; Pizzuto, 2007; Garcia and Sallandre, 2020). One key tenet of the semiological approach is the gaze behavior of the signer and the interlocutor, and how the interlocutor follows the signer’s eye gaze in concurrent with their signing. It is the coupling of the eye gaze with the pointing sign or the directional verb contributes to the meaning of reference in discourse.

In addition to the mental spaces, the neo-Peircean semiotic, and semiological approaches, there is the Places view. Wilcox and Occhino (2016), Wilcox and Martínez (2020) take issue with the indicating verbs analysis, stating that it relies on structuralist assumptions about the cognitive representation of language. These researchers point out that the categorization of sign and co-sign gesture suggests that language is composed of “discrete symbols and classical categories with strict boundaries” (Wilcox and Occhino, 2016, p. 26). The categorization also suggests that deaf signers and hearing speakers would share the same understanding about the structure of language, regardless of one’s access to language and experience with it. Wilcox and their collaborators adopt a Places view, which is strongly influenced by Cognitive Grammar (Langacker, 1987, 1991, 2008) and usage-based linguistics (Bybee, 2006, 2010), The Place is a “symbolic structure, a pairing of a meaning and a location in space” (Wilcox and Martínez, 2020, p. 2). On the surface, the mental spaces and the Place views appear very similar. But a major difference between these views is that the Place view does not consider the non-listability of locations to be a problem and instead views locations as schematic. The selection of the location would be motivated by a user’s sensory experiences with the world and abstraction from recurring usage events. Pointing constructions and directional verbs are viewed as complex symbolic structures composed of a pointing device and a Place. The pointing device “functions *to direct or focus attention* to the Place” (Wilcox and Occhino, 2016, p. 8), so a directional verb would direct attention to Places associated with the referents and profile a process. As the pointing device and the Place are already symbolic structures, and pointing constructions and directional verbs are also symbolic structures, verb agreement therefore is framed as *multiple symbolization*.

### A Proposal for a Usage-Based Analysis

Notwithstanding the extensive attention to directional verbs, there are numerous issues that have yet to be fully addressed. Directional verbs exhibit much morphophonological variation for marking person, which makes it challenging to generalize about the productivity what forms are “regular” and “irregular.” Some verbs like OWE cannot exhibit subject agreement due to articulatory constraints (Rathmann and Mathur, 2002). There are a handful of verbs that cannot mark first-person object forms (Hou and Meier, 2018). These issues make it a challenge to identify argument structure constructions of directional verbs in ASL, particularly from a usage-based perspective, in the absence of a large-scale, searchable corpus of ASL, though this has been done for Auslan (de Beuzeville et al., 2009; Johnston, 2019b). Usage-based linguistics posits that verbs are associated with specific argument structure constructions. The representation of these structures is said to be shaped by two general properties: (1) frequency of occurrence of verbs and (2) the meaning of words and constructions in use.

In lieu of corpus data, empirical observations of directional verbs in argument structure constructions are grounded in a sampling of internet data for forming a preliminary usage-based analysis. For the time being, I take a non-committal stance on the linguistic status of verb agreement by using ‘‘directional verbs’’ (this term may include ‘‘spatial verbs’’ but I do not discuss them here) and view ‘‘verb agreement’’ as a language-specific concept but distance myself from multiple aspects of the formalist views of agreement. I do assume that the verbs mark person, as reflected by the glossing practice of the examples from my data collection, though I keep an open mind to a wider interpretation of person marking based on the data. I do not assume that agreement in ASL patterns like agreement in other languages regardless of whether they are signed or spoken, and furthermore, what may look like agreement may not be agreement. Using alternative terminology like ‘‘indicating verbs’’ that is not used for describing spoken languages is unfortunately not very accessible to the wider field of linguistics and renders signed language research obscure to spoken language linguists^2^. Using more conventionalized terminology, however, does not preclude sign language linguists from proposing an alternative proposal to analyzing verbs. Researchers can be more explicit about what they mean by a concept and what kind of theoretical assumptions are packed in their analysis. Thus it is possible to use “agreement” as a comparative concept for typological purposes while describing the operationalization of agreement in language-specific terms (Haspelmath, 2010; Croft, 2016). This principle extends to signed languages as well (Lepic, 2021). So using “ASL agreement” is a starting point for developing a usage-based analysis of the argument structure of directional verbs in ASL, but this should not preclude an expanded and nuanced understanding of “ASL agreement” to the point where it may be eventually described in other comparative and language-specific terms.

## Data and Methods

The present study utilizes internet data. Some of the data has been previously analyzed in a study on first-person object forms of directional verbs (Hou and Meier, 2018) and another study about the functions of LOOK as a verb of visual perception and as a stance marker (Hou, 2022). Internet data refers to videos and vlogs in any signed language created and published by deaf signers on commercial social media platforms such as YouTube, Facebook, Instagram, and the likes. The rationale for using internet data is that there is no publicly available, machine-readable corpus for ASL yet (Morford and MacFarlane, 2003; Wilkinson, 2016; Lepic, 2019; Occhino et al., 2021). In lieu of an established corpus, the internet data is a suitable alternative (Hou et al., 2020, 2022). The U.S. variety of mainstream ASL is one of the most common signed languages represented on the internet, owing to the omnipresence of media sources such as the Daily Moth and DPAN.TV and widely shared public vlogs (Hou et al., 2020; Snoddon and De Meulder, 2020).

An issue with internet data (and data in general) is the frequency of signs in ASL. There have been only a couple small-scale studies that investigated the token frequency of occurrence for individual signs (Morford and MacFarlane, 2003; Mayberry et al., 2014; Sehyr et al., 2021). The limited sampling of data however means that these studies may not be wholly representative of the ASL lexicon. Still, these studies can indicate the frequency of occurrence of some signs. Morford and MacFarlane (2003) listed 37 signs that occurred more than four times per 1,000 signs in a database that consisted of 4,111 signs. The verbs TELL and LOOK, were listed among these signs. They had 7.1 occurrences and 6.3 occurrences per 1,000 signs, respectively. These findings align with what has been reported for BSL in Fenlon et al. (2018), who listed the frequency of 81 verb types of 1,436 verbs from narrative data in a considerably larger BSL corpus. Fenlon et al. reported the ten most frequent types in the following order: SAY, LOOK, LOOK2, GIVE, MEET, GIVE-INFORMATION, ASK, PAY, TEACH, and HELP.

One however must be careful about using reported frequency studies of verbs in other signed languages to speculate about the frequency occurrence of verbs in ASL. In a corpus study about the frequency and duration of signs and parts of speech in Swedish Sign Language (SSL), Börstell et al. (2016) listed the 300 most frequent types of signs in a sample of 44,786 signs. The ten most frequent types for verbs were TO-BE, HAVE, PERF, BE-INSIDE, TO-MEAN, SEE, TO-SIGN, REMEMBER, LOOK-AT, and COME-THERE^3^. As the researchers were interested in parts of speech, they did not distinguish the verbs based on their ability to participate in agreement. Therefore, for the purposes of this study, seven directional verbs were selected from two datasets: ASK, TELL, REMIND, AWARD, GIVE, CONVINCE, LOOK. The verbs were grouped for their semantic similarity: ASK, TELL, and REMIND for reported speech constructions, AWARD, GIVE, and CONVINCE for passive constructions, and LOOK for stance verb constructions.

The above seven verbs have been rated by deaf signers to have a moderate to high frequency of occurrence. Sehyr et al. (2021) discussed the signers’ online frequency rating of signs from ASL-LEX, a publicly available, large-scale lexical database for ASL. The current version of ASL-LEX contains 2,723 signs. The signers were asked to rate different signs based on their intuition about the frequency of occurrence in everyday conversation, using a 7-point scale in which 1 indicated “very infrequently” and 7 indicated “very frequently.” The signs were ranked in the following order of increased frequency: CONVINCE (4.036), REMIND (4.9), CALL (4.967), LOOK (5.08), AWARD (5.222), ASK (5.24), GIVE (5.667), TELL (5.933). These subjective ratings somewhat correlate with the corpus estimates, although the ratings do not distinguish lexical categories of the signs.

For all the seven signs, I selected the tokens that distinctly functioned as verbs, rather as nouns, for example, based on the sign’s position and role in the utterance. All these verbs can be spatially modified to mark what look like first-person and non-first person object, though they vary in degree of reported frequency. The first dataset is for reported speech and passive constructions. There are seven videos totaling 1 h and 24 min with a total of 145 verb tokens. Most videos are monologs or live narratives. One video is a news report from the ASL radio show, *The Daily Moth.* The second dataset is for stance verb constructions with LOOK tokens used in Hou’s (2022) study. There are 65 videos totaling 8 h 21 min with a total of 349 verb tokens. The videos encompass a more variety of genres, though dyadic and polyadic conversations are underrepresented. Both datasets total to 494 verb tokens; they overlap for the most part except for Video 5. This video was specifically selected for the repeated occurrence of GIVE and AWARD. Table 1 shows the summary of the first dataset, and Supplementary Appendix 1 lists the video sources for both datasets.

**TABLE 1:** Summary of the verb tokens in the first dataset (n = 145).

Video source	GIVE	TELL	AWARD	CONVINCE	REMIND	LOOK
Video 1	0	4	0	0	1	22
Video 2	0	2	0	0	3	30
Video 3	0	0	0	2	0	18
Video 4	0	0	0	1	0	3
Video 5	18	6	5	0	1	1
Video 6	0	2	0	0	3	3
Video 7	0	2	0	0	1	9
	*18*	*14*	*6*	*3*	*8*	*86*

### Analysis

The data was coded in ELAN. The verb forms were coded for person and syntactic and semantic arguments based on the discourse context. There has been discussion about how to judge whether a directional verb is modified or not (de Beuzeville et al., 2009; Fenlon et al., 2018), but the criteria is based on the concept of a citation form of a verb. A verb form could be “unmodified” or “congruent” but could be compatible with a modified interpretation. For example, a verb form precedes a non-localized object argument, i.e., an utterance like TELL PEOPLE, does not have PEOPLE localized in space. Another example is that a verb form targets a second-person object argument and may resemble the citation form. Such ambiguous forms occurred in the data. Only one clear exception was when a verb form was not modified to mark a first-person object argument and was followed by a first-person pronoun; the form moved away from the signer instead of moving toward the signer, which appeared to understate the event of looking. Some LOOK tokens exhibited reduction in the modification, which I attribute to the ongoing grammaticalization of the verb as a visual perception verb to a stance marker. Overall, I decided that judging the spatial modification of verb forms for whether they were “unmodified” or “congruent” could lead to a deep rabbit hole, and moreover, spatial modification was not the sole contributor to the interpretation of the argument structure constructions. I only judged a verb form to be unmodified when there was a clear case like the above first-person object argument situation.

Many scholars have observed that there is a strong interplay between constructed action (CA) and the spatial modification of directional verbs in signed languages, i.e., a verb is more likely to be clearly modified during CA (de Beuzeville et al., 2009; Jantunen, 2017; Fenlon et al., 2018; Hodge and Cormier, 2019; Johnston, 2019b). CA is generally described as a stretch of discourse, of any length, that represents a role of a referent other than the present narrator or roles of multiple referents whose actions, thoughts, or feelings are depicted (Cormier et al., 2015b). The depiction emerges through hands, face, and other parts of the signer’s body; the narrator can depict the referent by “telling” with signs and/or “showing” with their body and vary in the degree of saliency. CA has been described as a form of gestural enactment that represents the semiotic diversity of linguistic expressions. I adopted some of Cormier et al. (2015b)’s proposed criteria for coding a subset of the data – verbs of communication – for the occurrence of CA, since I observed that these verbs exhibited more explicit CA than the other verbs in the dataset. I relied on the changes of the facial configuration and eye gaze as well as shifts in body positioning. CA can include constructed dialogue (CD), which has been reported to co-occur or overlap with CA and involves the direct or indirect quotation, or perhaps both. For the sake of parsimony, I chose not to tease apart CA with CD from CA without CD or CD without CA, due to the small size of the data sampling in the present study. Moreover, I avoid positing constraints on the occurrence of CA in the proposed argument structure constructions since CA varies widely in its distribution and degree of explicitness in discourse (Cormier et al., 2015b; Jantunen, 2017; Koulidobrova and Davidson, 2020).

For this paper, the boundaries of clauses of all the argument structure constructions are not explicitly delineated. The complexity and ambiguity of identifying the clause boundaries of spontaneous signing in line with the Auslan corpus annotation guidelines (Johnston, 2019a) require extensive labor that requires more than that of an individual researcher available. The eye-gaze behavior is not explicitly delineated, either. Much of the internet data does not involve live shared eyegaze between the signer and the audience, which presents a somewhat novel problem for the interpretation of the signer’s eyegaze. Thus, the proposed templates for argument structure constructions should not be interpreted to mark clausal boundaries.

## Argument Structure Constructions

### Reported Speech Constructions

A reported speech construction involves verbs of communication used to report the speech of a person directly or indirectly^4^. ASL has a large group of directional and non-directional verbs of communication that may be used for constructed dialog such as ANNOUNCE, ASK, BAWL-OUT, CALL-BY-PHONE, INFORM, INSULT, MAKE-FUN, MOCK, ORDER, SAY, REMIND, TEASE, TELL, and WARN. The selected three verbs of communication are TELL, ASK and REMIND. These glosses represent the approximate meaning of the verbs, though REMIND primarily is associated with the meaning of bringing someone’s attention to something that requires action.

I propose that there are two overarching types of recurring reported speech construction (hereafter RSC) that the three verbs participate in. Table 2 presents the summary of the distribution of the verbs of communication across the RSC types. In the following written examples, for the sake of space, CA is indicated by the brackets [CA:]. Non-manual expressions are not included.

**TABLE 2:** Count summary of recurring RSC Types for the three verbs of communication.

*Reported speech construction types*	Tell (n = 16)	Ask (n = 7)	Remind (n = 9)	*Total* (n = 32)
Type 1	15	6	9	*28*
Type 2	1	1	0	*2*


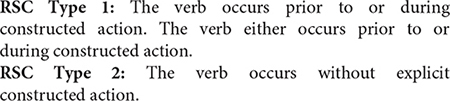


There are 16 tokens of TELL. All tokens except one appeared to be spatially modified for non-first person object arguments. All tokens of TELL except for one occur in RSC Type 1. In (6) and (7), TELL occurs prior to or during constructed action, as marked by the brackets. In (8), which is the only token that fits RSC Type 2, there is no explicit occurrence of CA, which can be attributed to the example presented as a comment about the story that the narrator was telling the audience.


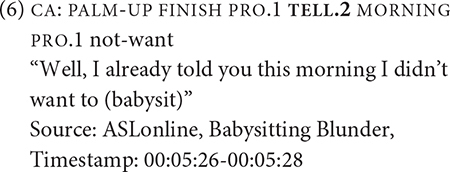



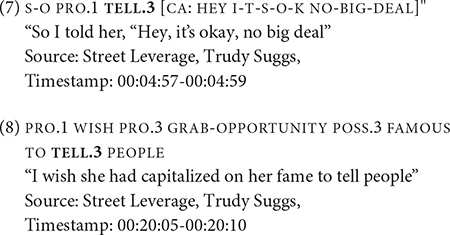


**FIGURE 2:**
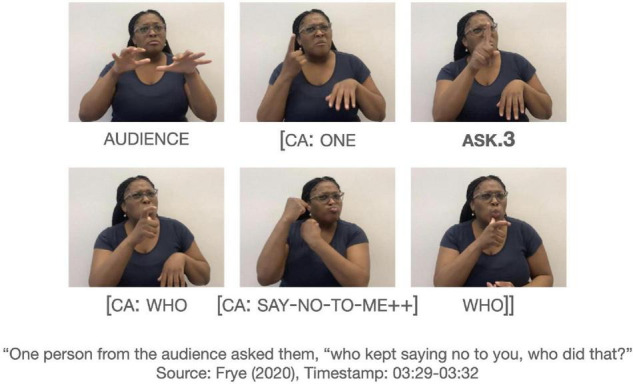
An example of ASK in RSC Type 1. Images produced with permission, source information available in the Supplementary Material.

For the seven tokens of ASK, all tokens of ASK except one appeared to be spatially modified for non-first person object arguments. Three tokens were modified for repetition, as indicated by “++,” giving the reading of “to question” rather than “to ask a question.” Six tokens occurred in RSC Type 1. Figure 2 shows the occurrence of ASK in RSC Type 1; this example contains complex, nested constructed action. In this figure, during the production of ONE, the narrator’s face and eye gaze changes, signaling a transition to constructed action to enact a referent. Following ASK.3, the signer starts quoting the first referent’s question to a second referent, WHO, and then quotes a third referent who said SAY-NO-TO-ME + + from the second referent’s perspective. Basically, the first referent is quoting what was being told to the second referent.

Example (9) represents one token of ASK that corresponds to RSC Type 2. In this example, the narrator recounts about being asked many questions, as indicated by the symbols + +, but does not quote or even paraphrase what the questions were.


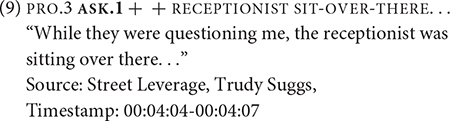


For the 9 tokens of REMIND, six tokens were modified for non-first person object arguments and three for first-person object arguments. All tokens of REMIND occurred in RSC Type 1, as shown in (10) and (11). Note that (10) also exhibits complex, nested CA, as the signer mouths YOU HAVE THREAD and subsequently enacts the thread hanging from their own butt.


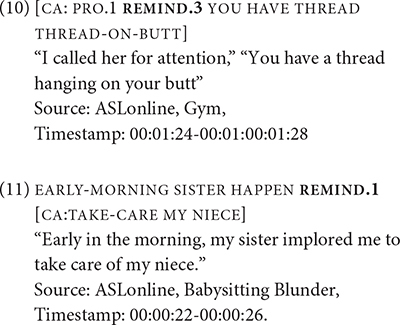


What tentative generalizations can be abstracted from the above data? First, most verb tokens occur in RSC Type 1, suggesting that there is a strong interaction about directional verbs of communication and constructed action. These verb tokens tend to occur during CA or prior to the inception of CA. Second, very few verb tokens occur in RSC Type 2, suggesting that CA is, more or less, integral to argument structure constructions of these verbs of communication. Based on these findings, the following constructions are proposed to capture the various reported speech construction types. The paucity of the data does not allow for clear generalizations about the frequency of present and absent of subject and object arguments, so for the time being, I posit that these arguments are optional.


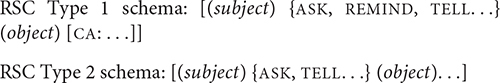


Given the wide and varied distribution of CA in the grammar of many signed languages (Cormier et al., 2015b; Jantunen, 2017), it may be challenging to posit where exactly CA occurs in argument structure constructions. If one is concerned with parsimony, one could propose templatic constructions without CA. Garcia and Sallandre (2020) state that the semiological approach does not follow the CA model, as most approaches in sign language linguistics do, but instead categorize CA as part of the ‘highly iconic constructions^5^.” Alternatively, one could draw more clear distinctions between different verb types and CA, if one has an adequate dataset for abstracting generalizations.

### Non-dedicated Passive and Reflexive Constructions

Another type of argument structure construction of interest is the passive construction. This generally refers to defocusing the agent, which is the main pragmatic function of passives (Shibatani, 1985). Defocusing the agent may occur by omitting the agent or not specifying it in various syntactic and morphological ways. In the dataset, I identified a few verb tokens, AWARD, CONVINCE, and GIVE that occur in both active and passive constructions, though a few tokens of the latter warrant further scrutiny. Table 3 summaries the distribution of the three verbs for active and passive constructions. Most verb tokens are modified for third-person object arguments only, though the tokens of GIVE are modified for both subject and object arguments, all third-person. Only one token of AWARD and three tokens of CONVINCE are modified for first-person object arguments; these tokens occur in passive constructions. Three tentative types of passive constructions are proposed, following a short discussion on the formational differences between active and passive constructions and an examination of aggregated data from previous and current research.

**TABLE 3:** Count summary of active and passive/reflexive construction types for three verbs.

	Give (n = 18)	AWARD (N = 6)	CONVINCE (N = 3)	*Total* (n = 29)
Active construction	1	1	0	*5*
Passive/reflexive construction	17	4	3	*24*

There have been various proposals for how different signed languages express “passive constructions,” which encompass R-impersonals and agent defocusing (Janzen et al., 2001; Leeson, 2001; Rankin, 2013; Barberà and Cabredo Hofherr, 2017; Nordlund, 2019). R-impersonal constructions have subjects that are human and non-referential such as impersonal subject pronouns (Siewierska, 2011). The current consensus is that passive constructions can be semantically marked but not morphologically or syntactically marked, since there is no change in the verb form and the object is not promoted to the subject position. The corollary is that such constructions can give indefinite readings; this is further discussed below. Signed languages therefore lack “dedicated” passive constructions, hence the “non-dedicated passive” title of this section.

In the case of directional verbs, there is no change in the direction of path movement and palm orientation. Consider GIVE, a highly cited verb for its morphological “versatility,” i.e., it can be spatially modified to point to different locations, including the signer. In many signed languages GIVE exhibits strong transitivity for its three-argument verb structure (Börstell et al., 2019). The ASL GIVE is no exception to this generalization. Examples (12) through (15) shows GIVE in several double object-like constructions from Padden (1988). In (12) through (15), there is an explicit subject/agent, INDEX, and theme, BOOK. The verb form contains subject and object agreement markers, which are indicated by the subscripts (“1” for first-person, “2” for second-person, and “i” for index for third-person) attached to the gloss GIVE. The recipient may be an actual referent in which the real-world location serves as the final location of GIVE, or it may be based on an arbitrary location in the signing space and that location is already associated with the referent in prior discourse. These examples suggest that the ditransitive construction appears to be a prototypical representation of the argument structure of GIVE.


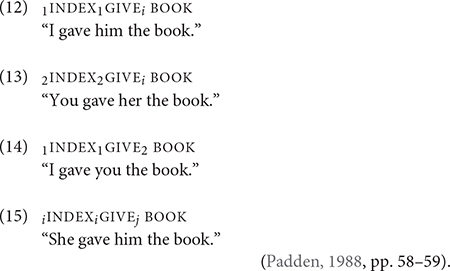


(Padden, 1988, pp. 58–59).

In (16) and (17), which are from Padden (1988), the subject agreement marker, indicated by the “0” subscript, can be omitted. The initial location of the verb is not associated with the subject. But the omission of the subject agreement marker does not alter the fundamental meaning of transitivity if there is an independent nominal (or pronominal) for an explicit agent in (16). Otherwise, the absence of an explicit agent in (17) and its English translation suggests either the example may be either an impersonal or passive construction. The ambiguous translation shows that both meanings are possible, though if there was an impersonal subject present, the example would give a stronger reading of an impersonal construction.


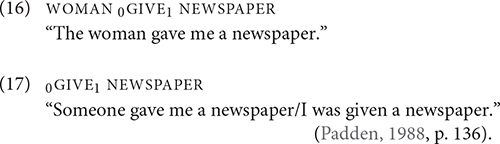


(Padden, 1988, p. 136).

Janzen et al. (2001) proposed a distinction between active and passive constructions in ASL. In their view, a passive construction is formed by the configuration of various grammatical features, including the use of constructed action and the defocusing of an agent in the clause. A prototypical passive construction foregrounds point of view of the patient (or the recipient) through constructed action while defocusing the agent (Janzen et al., 2001; Rankin, 2013). The verb form however does not change when it occurs in a passive construction, e.g., omitting the agent or demoting it to an oblique argument and promoting the patient to the subject argument. (18) shows the spatial modification of a two-handed form of GIVE, as indicated by “(2 h),” from a non-first person agent to a first-person recipient, which are represented as “a” and “1” in subscripts, respectively. Yet there is no explicitly identified agent. The only explicit argument is the basketball tournament, and it functions more as a theme than as an agent. Although the movement of the verb form implies an agent giving the basketball team a trophy from a first-person perspective, the agent is not specified and therefore is not referential.


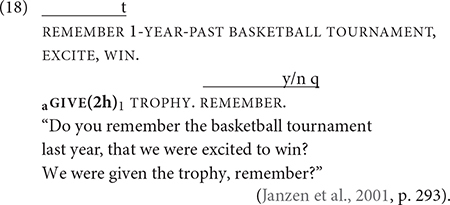


(Janzen et al., 2001, p. 293).

Rankin (2013) argues that the omission of the agent *without* constructed action is the most common strategy for passive constructions with non-first person patients as shown in (19); the superscript ^→*right*^ indicates the direction of the verb, so as to distance from referential indices. Constructed action, then, does not appear to be an obligatory element of passive constructions in ASL.


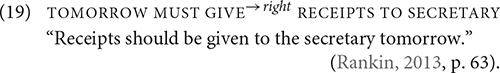


(Rankin, 2013, p. 63).

The internet data corroborates the findings of Janzen et al. and Rankin. The above examples involve first-person object forms of GIVE that co-occur with and without constructed action, so I present a different example of GIVE with non-first-person interpretations in 20, which is the same as Figure 1. This example does not exhibit any elements of constructed action. The verb form can be interpreted to indicate the transfer of an object from a non-first person agent to a non-first person recipient. This interpretation is based on the initial and final locations of the verb: the verb starts at a spatial location to the signer’s right and moves toward to another location ahead of the signer’s chest. There is no explicitly identified agent, and prior to the clause, the signer was narrating about how one movie was erroneously announced as the winner of an award. This event is memorialized as “Envelopegate.” The example marks the beginning of a long description of the snafu with the envelopes, indicated by the signer’s calling attention with HEY and the co-occurrence of raised eyebrows with WHAT.DO HAPPEN as a rhetorical strategy of offering new information. There is a total of 18 tokens of GIVE in the entire discourse about the “Envelopegate.” All tokens, with one exception, occur in a semantically passive construction; the last token co-refers to a newly identified agent.


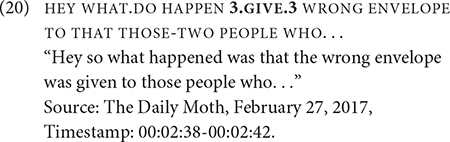


What remains an open question is the number and type of directional verbs that can participate in similar non-dedicated passive constructions as GIVE. Another verb, AWARD, also denotes the transfer of an object to a recipient, except the object is specifically an award. Out of the six tokens of AWARD in the dataset, one only occurred in an active construction, specifically a ditransitive construction in (21).


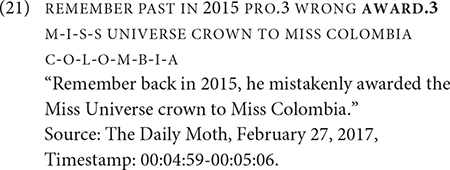


Five AWARD tokens occur in a non-dedicated passive construction. One such token occurs in Example (22), in which the agent is omitted; there is no identification of the agent in prior discourse. The recipient occurs preverbally in the subject. Two other tokens have the recipient in a similar position and one token has the recipient in a post-verbal position.


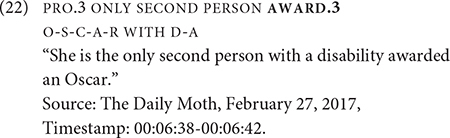


**FIGURE 3:**
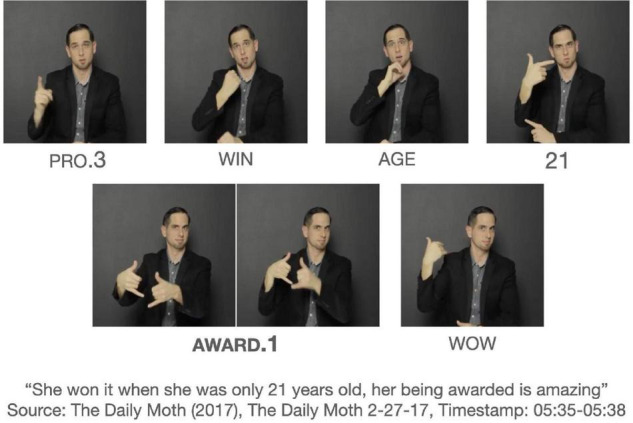
An instance of AWARD.1 in an apparently non-first person reading. Images produced with permission, source information available in the Supplementary Material.

There is one more AWARD token that does not fit the structure of the other passive construction. This token is spatially modified for the first-person object argument. Figure 3 shows AWARD.1 without the identification of an explicit agent in the discourse, and the initial location of the verb is not associated with a specific agent. This example stands out for the first-person object verb form, since (22) shows a non-first person object form targeted at the referent, who is represented on the signer’s right side. This is also shown by the occurrence of PRO.3 in Figure 3. There appears to be two clauses as delineated by the brackets: [PRO.3 WIN AGE 21] [AWARD.1 WOW]. The AWARD.1 occurs in the second clause. Given the context and clausal boundaries, AWARD.1 does not mean “I was awarded” but rather emphasizes the reception of the award with the signer’s body, given its strong association with the signer’s body as first person (Meir et al., 2007). Thus, the example in Figure 3 may be a verbless attribute clause^6^.

**FIGURE 4:**
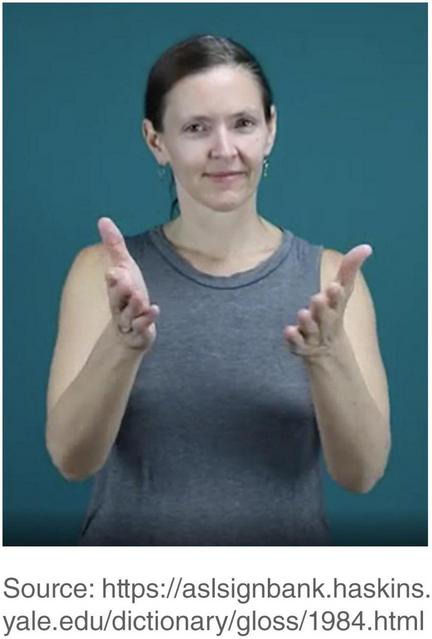
The dictionary entry for CONVINCE. Source: https://aslsignbank.haskins.yale.edu/dictionary/gloss/1984.html. Images produced with permission, source information available in the Supplementary Material.

For CONVINCE, the passive constructions with this verb give a different reading than the ones with GIVE and AWARD. Figure 4 is the dictionary entry for CONVINCE. It is listed as an “inflectional” verb in Padden (1988) and an “agreement” verb Mathur (2000). It is noted to have an “idiosyncratic first-person object form” which arguably must be listed because this particular form is produced on both sides of the signer’s neck rather than the signer’s chest (Meier, 1990; Lillo-Martin and Meier, 2011; Hou and Meier, 2018). The chest is the most common location for the end point of first-person object forms of directional verbs, so the neck is marked for being the location for the end point of CONVINCE-1. No other directional verb has been documented to occur on the sides of the neck, although a few other signs do occur in the proximity of that location, such ‘‘bankrupt’’,^7^ ‘‘vampire,’’^8^ and ‘‘accent’’^9^.

**FIGURE 5:**
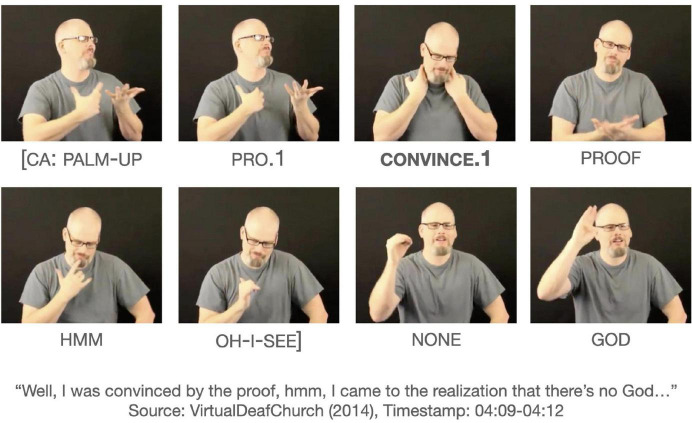
An example of in CONVINCE.1 in an indefinite construction. Images produced with permission, source information available in the Supplementary Material.

To date, there has been virtually no examples for CONVINCE. The internet data revealed only three tokens of CONVINCE, all modified for first-person object arguments. Figure 5 shows the occurrence of CONVINCE.1, taken from a narrative about a signer’s journey to atheism. Prior to this clause, the signer narrated about joining a group for skeptical pastors who had long, agonizing conversations about the existence of God. These conversations eventually led the signer to reach a conclusion. The verb form CONVINCE.1 alone could mean ‘‘they convinced me’’ in which the agent refers to the group. However, unlike GIVE, the initial location of CONVINCE does not clearly indicate the agent. There is a PALM-UP (an interjection meaning ‘‘well’’ here) and a first-person pronoun preceding CONVINCE.1 and PROOF following it without a discernible break. An alternative interpretation is that the verb has a self-reflexive reading as in, ‘‘I convinced myself with the proof^10^.” The signs CONVINCE.1 and PROOF co-occur with similar head movement, suggesting that they occur within the same clause. Because PROOF appears to be part of the clause, functioning as an adjunct argument, it is difficult to determine whether the example in Figure 5 is a non-dedicated passive construction or a reflexive construction.

**FIGURE 6:**
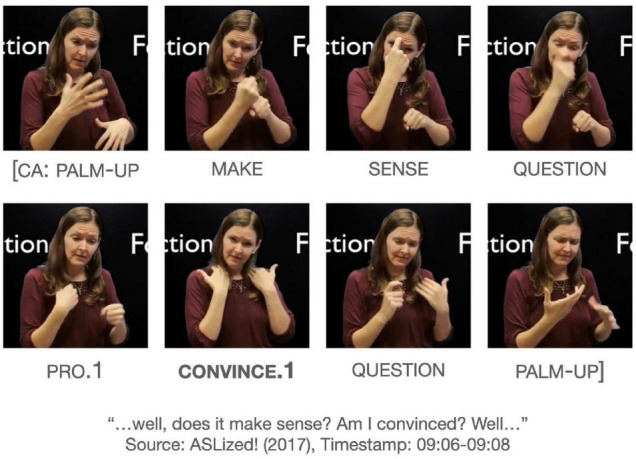
Another example of CONVINCE.1 in a passive construction. Images produced with permission, source information available in the Supplementary Material.

Another example with the same string of signs of PRO.1 CONVINCE.1 and a similar interpretation occurs in Figure 6. The context of the utterance is about reading an academic paper and figuring out whether the argument in the paper is intelligible. The signer is asking if one finds the paper convincing, rather than asking if one convinces oneself. Again, the most appropriate interpretation would be, “Am I convinced (by the paper)?” or “Do I find this paper convincing?” The signer later produces another token of CONVINCE.1 for the same interpretation.

Interestingly enough, there were another three tokens of CONVINCE + ONE, all modified for non-first person object arguments in the same video for Figure 6. CONVINCE + ONE is a related verb construction, in which a one-handed version co-occurs with a non-dominant extended index finger. Some directional verbs have similar constructions, e.g., GIVE + ONE and REMIND + ONE, since they all use ONE. Other non-directional verbs also overlap with these constructions, e.g., FLATTER^11^ and FOOL^12^. The function of ONE appears to mark affectedness, i.e., the object/patient is affected by the event. It remains an open question about the frequency of different forms of CONVINCE and CONVINCE + ONE in different argument structure constructions.

The paucity of the verb tokens makes it difficult to make a generalization about non-dedicated (and reflexive) constructions, so I propose several low-level constructions for GIVE.1, GIVE.3, AWARD.3 and CONVINCE.1 based on the aggregated data. The passive construction Type 1 and Type 2 differs by the verb form and the transitivity. First, the grammatical configuration of the syntactic positions appears to be shaped by the spatial modification of the verb for first-person and non-first person object arguments. Second, Type 1 is transitive but not ditransitive, whereas Type 2 is ditransitive for the occurrence of the direct and indirect object arguments and the introduction of the recipient by the sign TO.


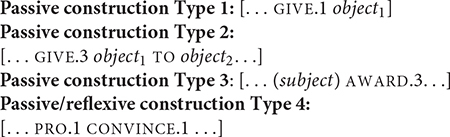


### Stance Verb Constructions

The third argument structure construction is the stance verb construction. This construction contains a verb that represents the user’s (inter)subjective positioning toward a situation. The definition of stance is vast and varied (Englebretson, 2007). It can range physical embodied action to epistemic modality to social morality, and researchers vary in their approach to stance and how they understand it. Some scholars have argued that stance can be identified across whole phrases in discourse, rather than individual words only, since a word can have many functions based on its relationship with discourse (Hunston, 2007; Wang et al., 2021). English has been documented and described for complement-taking predicates such as *know, think, guess, doubt, hope*, or *wish* (Kärkkäinen, 2003). These verbs represent different types of stance relating to knowledge and certainty, and can have a positive clause or a negative one in its scope (Dancygier, 2012). A statement reading *I think she’s there* signals a positive or affective stance, but another statement like *I think she’s not there* signals a more neutral stance, which comes from the occurrence of the negator “not.” There is extensive literature on stance as expressed lexically and grammatically in English and some other spoken languages, but not as much literature on signed languages. To date, research on stance in signed languages has been largely concentrated on modal verbs (Shaffer and Janzen, 2015).

The current focus is the stance function of a sign, LOOK, that collocate with a few signs, forming fixed and schematic multi-word expressions. LOOK is a sign visual perception that can be spatially modified for different types of meaning based on with path and manner of movement, the number of hands and configuration of the hands, and the type of facial expressions (Klima and Bellugi, 1979; Naughton, 2001). This sign has been generally considered an agreeing/indicating verb, though it does not always denote a verb of transfer between two animate arguments, since the targeted stimulus does not have to be animate (Mathur and Rathmann, 2012). The sign may or may not be transitive. A recent typological study showed that signs with the basic meaning of “to look at” in five signed languages rank lower than spoken languages on the scale of transitivity prominence (Börstell et al., 2019). Although ASL is not part of Börstell et al.’s study, a similar pattern of transitivity prominence is observed in the LOOK data. The LOOK sign has two broad functions, vision and reaction. LOOK/“vision” functions as a directional verb that targets numerous animate and inanimate objects; in other contexts, it functions as an intransitive verb or as a noun. LOOK/“reaction” functions as a stance marker that conveys the signer’s reaction toward a sensory, usually visual, stimulus (Hou, 2022). The reaction tends to occur in the form of a quotative or exclamatory statement. It is interpreted as “be + like,” similar to what Padden (1986), Lillo-Martin (1995) used for translating quotative and non-quotative constructions in ASL to English. This interpretation also echoes the grammaticalized English “like” to introduce reported speech and thoughts (Romaine and Lange, 1991).

The two functions are distinguished by the multi-sign sequences which they participate in. Hou conducted a preliminary study of *n*-grams, a contiguous sequence of identifiable signs, that repeatedly co-occurred with LOOK/“vision” and LOOK/“reaction” (Hou, 2022). The sequence was considered recurring if a string of two or more signs occurred at least two times in the dataset. The sequence included at least one sign that precede and/or follow the verb. The dataset consists of 8 h 21 min of 64 videos by 38 unique deaf signers, which yielded 706 tokens belonging to the family of “look” signs. These tokens include OBSERVE, READ, and PERSPECTIVE, as they share the V-handshape and the visual perception meaning, so the LOOK/“vision” function is not limited to one sign. Some tokens are ambiguous in the sense that the function of a token simultaneously exhibits vision and reaction or overlaps with both vision and reaction. In some instances, the function is unclear. An example would be the co-occurrence of READ followed by a reaction. Table 4 summarizes the number of tokens and types for both functions and the ambiguous tokens. Table 5 summarizes the frequent bigrams, trigrams, and quadgrams for the “look” signs by function.

**TABLE 4:** Summary of the family of “LOOK” signs by function, token, and type.

Function	Token Count	Type Count
Reaction	174	1
Vision	369	18
Ambiguous	163	17
*Total*	*706*	*36*

**TABLE 5:** Summary of frequent (n ≥ 2) n-grams of the “look” signs.

Function	Bigrams	Trigrams	Quadgrams
Reaction	38	24	4
Vision	77	13	2
Ambiguous	28	5	0
*Total*	*143*	*42*	*6*

There are multiple recurring sequences observed in the 174 tokens of LOOK/“reaction.” Table 6 summarizes the 38 recurring bigrams for these tokens. The “s” is short for “sign,” representing LOOK. The “s-1” represents the sign preceding LOOK while “s + 1,” and “s + 2” represent following LOOK, respectively. Some signs recur in the s-1 or the s + 1 slot and have similar counts, so they are grouped together. What Table 6 shows that 55% of the bigrams have PRO.1 in the s-1 slot and 21% have OIC (short for OH.I.SEE) in the s + 1 slot. Apart from PRO.1, various non-first person pronouns and referents occur in the s-1 slot. Apart from OIC, a handful of specific “reaction” signs such as PALM-UP an interjection meaning “well,” GET.INSPIRED, HOLD.ON, MIND.PUZZLED, WOW, and FINE in the s + 1 slot. The rest of tokens collocate with hapaxes such as WHAT’S.UP, STOMACH.TURN, and CONCERNED. These low-frequency sequences reflect the schematic use of the specific argument structure construction for LOOK/“reaction.”

**TABLE 6:** Frequent (n ≥ 2) bigrams in 174 tokens of LOOK/“reaction.”

Rank	s–1	s + 1	Count	
1	PRO.1		95	55%
2		OIC	36	21%
3		PRO.1	13	7%
4	PRO.3		11	6%
5		PALM.UP	10	6%
6	PEOPLE		8	5%
7	PALM.UP	WOW, YES	5	3%
8		FINE, GET.INSPIRED, HOLD.ON, MIND.PUZZLED	4	2%
9	DEAF, PRO. 2, SIGN.FLUENTLY	PRO.3, QUESTION, REALLY, THINK, WAVE.NO, WONDER	3	2%
10	MAYBE, WOMAN, WILL, SECRETARY	AWFUL, BE.FASCINATED, CAN’T, DISMISS, FEEL, GUT.INSTINCT, HOW, NO, NONE, THAT, THESE.TWO, THINKING.HARD	3	1%

Table 7 summarizes the 40 recurring bigrams for the 150 tokens of one type of LOOK/‘‘vision’’ (this type is glossed as LOOK). Although PRO.1 is the most frequent sign to collocate with LOOK/‘‘vision,’’ PRO.1 only accounts for 15% of the 150 tokens^13^. There is a larger distribution of various signs collocating with LOOK/“vision” which includes various modals, negators, and nouns, possibility reflecting the schematicity of a different argument structure construction. None of these signs group together as a category that would distinctly signal to the signer’s reaction to a visual stimulus, even when considered in the larger context of discourse.

**TABLE 7:** Frequent (n ≥ 2) bigrams with LOOK/“vision” (n = 150).

Rank	s–1	s + 1	Count	
1	PRO.1		23	51%
2	PALM.UP		9	6%
3		PALM.UP	9	6%
4	CAN, INDEX	PRO.1	6	4%
5	PRO.2	SEE	5	3%
6		INDEX	4	3%
7	NEVER, O-R	SUN, THAT	3	2%
8	CAN’T, FINE, GRAB.OPPORTUNITY, HAVE.TO, LOOK, MUST, NOT, NOT.YET, NOW, PICK.UP, START, TEND.TO, PRO.3-PL, WAIT, WILL, PRO.2-PL	FOLLOW, LOOK, ON, POSS.1-PL, POSS.3, Q-U-A-L-I-T-Y, T-V, V-I-D-E-O, WORD, Y-O-U-T-U-B-E, POSS.2	2	1%

**FIGURE 7:**
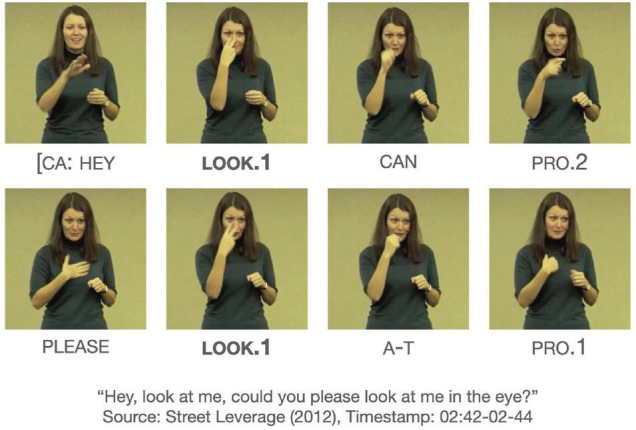
An instance of LOOK/“vision.” Images produced with permission, source information available in the Supplementary Material.


Figure 7 exhibits an utterance with two tokens of LOOK.1/“vision,” both clearly modified for first-person object arguments. The whole utterance co-occurs with CA, and the string of LOOK.1 AT PRO.1 is attested to have two tokens in the dataset.

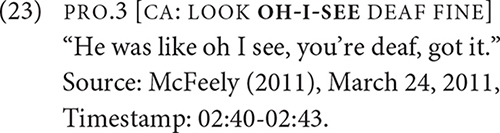


Exhibits an utterance with a token of LOOK/‘‘reaction,’’ which is followed by a statement that shows the signer’s positive stance toward the deaf person^14^. Both utterances in Figure 7 and example (23) co-occur with CA, so the LOOK/“reaction” cannot be viewed strictly as a CA phenomenon. What distinguishes the utterances is the meaning that emerges from the collocation of certain signs with LOOK. The semantic roles of the subject are not necessarily the same. The subject of LOOK/“vision” is an agent who directs their eye gaze at a visual stimulus whereas the subject of LOOK/“reaction” is more closely aligned with an experiencer who processes the visual stimulus.

Additionally, there appears to be some formational differences of the verb among the two functions. Compared to LOOK/“vision,” many tokens of LOOK/“reaction” exhibit less directional path movement, which may be indicative of phonetic reduction as part of an ongoing grammaticalization of the verb. In Figure 7, the verb form moves toward the signer’s own face, but in (23), the verb form does not exhibit as much as path movement. The formational differences associated with the functions warrant further investigation.

The data for LOOK/“vision” and LOOK/“reaction” provides evidence for the differences in argument structure constructions associated with the functions. LOOK/“reaction” is more syntactically restricted than the former, occurring in more fixed sequences such as LOOK OIC. At the same time, these sequences allow for the instantiation of a more schematic template, allowing for low-level constructions with slots that can be filled with other signs for the positions of the subject and the reaction. The following constructions for the two LOOK functions are proposed:


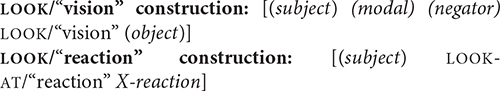


### Recapitulation

What does the usage-based linguistics approach to argument structure constructions of directional verbs do for the controversy? The existence of multiple theoretical frameworks indicates that the controversy may never be entirely resolved, unless sign language linguists can put their views aside and “come to an agreement on how to segment sequences,” including non-manual elements, for marking reference (Garcia and Sallandre, 2020, p. 15) for cross-linguistic purposes of comparing the structure of different signed languages. What I have shown here is that one can go beyond looking at the spatial modification of the directional verb and focus on the function of the verb based on the larger construction of the discourse. Although the data presented here is not comprehensively annotated and analyzed in line with the Auslan corpus annotation guidelines (Johnston, 2019a), the data does show how directional verbs function more than just marking pronominal reference and spatial transfer of objects.

## Future Directions

This paper has advocated for a usage-based approach to analyzing argument structure constructions of directional verbs in ASL as a way of identifying some of the grammatical patterns that make up a user’s linguistic knowledge. Seven verbs, ASK, TELL, REMIND, AWARD, CONVINCE, GIVE, and LOOK, were sampled from internet data and analyzed for argument structure constructions that they recurred in. The preliminary analysis revealed likely patterns for low-level constructions: reported speech constructions (ASK, TELL, REMIND), non-dedicated passive and reflexive constructions (AWARD, CONVINCE, GIVE), and stance verb constructions (LOOK). In reported speech constructions, it was shown that most tokens of verbs of communication occur in a construction that involved constructed action, whereas few tokens occur in a construction without explicit constructed action. For passive constructions, it was shown that many tokens AWARD and GIVE occur in agent defocusing constructions but one specific verb form of CONVINCE occurs in an indefinite construction that may be either non-dedicated passive or reflexive constructions. Finally, the stance verb constructions of LOOK reveal that it is the whole argument structure construction, not the verb itself, that give rise to the functions of vision and reaction.

Future research would look at a larger dataset of directional and non-directional verbs, allowing for more fine-grained generalizations about argument structure constructions. It has been many decades since the field of sign language linguistics became fascinated with verbs. With the advent of corpus and internet data, researchers are now in a position where they can abstract away from the controversy of verb agreement and to look for verbs in argument structure constructions, potentially advancing the field to a more holistic but deeper understanding of the interplay of language use and structure.

## Author Contributions

LH was responsible for every aspect of the contribution for the article, contributed to the whole conception and design of the study, wrote the entire manuscript and Supplementary Appendix, did the revisions, selected, transcribed, and organized the internet data in ASL, analyzed and discussed it thoroughly, and designed and edited all the figures.

## Conflict of Interest

The author declares that the research was conducted in the absence of any commercial or financial relationships that could be construed as a potential conflict of interest.

## Publisher’s Note

All claims expressed in this article are solely those of the authors and do not necessarily represent those of their affiliated organizations, or those of the publisher, the editors and the reviewers. Any product that may be evaluated in this article, or claim that may be made by its manufacturer, is not guaranteed or endorsed by the publisher.

## Footnotes

1 This is not to exclude classifier constructions, which also has received substantial attention (Zwitserlood, 2012).

2 Some spoken language linguists do reject the formalist views of agreement (Barlow, 1999; Croft, 2001, 2013; Langacker, 2008; Haspelmath, 2013; Kibrik, 2019). This is reflected in the usage of alternative but also conventionalized terminology such as *multiple symbolization* (Langacker, 2008) and *indexical coded dependencies* (Croft, 2001).

4 Reported speech also can occur in the form of depiction of actions, characters, and events through enactment in both signed and spoken languages, which suggests that reported speech is not a dedicated syntactic domain (Hodge and Cormier, 2019). That is, a user does not need to use conventionalized quotative markers or verbs of communication to express reported speech.

5 In some other approaches, “highly iconic constructions” refer to “non-lexical” (including CA) and “partly lexical” signs (Hodge et al., 2019).

6 I thank one reviewer for raising this excellent proposal. They suggested that AWARD.1 may be a non-finite verb that functions as the subject of the clause, but there is no evidence of any signed language containing morphology to distinguish finite verb forms from non-finite ones. Alternatively, AWARD.1 may be nominalized and thus would not be participating in verb agreement.

6 The sign BANKRUPT is a homonym: https://aslsignbank.haskins.yale.edu/dictionary/gloss/2607.html

8 The sign VAMPIRE is one-handed that occurs on the ipsilateral side of the neck: https://aslsignbank.haskins.yale.edu/dictionary/gloss/1371.html

9 The sign ACCENT is one-handed that occurs on the ipsilateral side of the neck: https://aslsignbank.haskins.yale.edu/dictionary/gloss/584.html

10 Shibatani (1985) mentions that in several Indo-European and American Indigenous languages, there is a morphological correlation between passive, reflexive, and reciprocal constructions, i.e., the same morphology is used for these two or three constructions. Since first-person object forms of directional verbs can be used in passive constructions (with the agent defocused), this raises the question about whether they can be used in reflexive constructions too. It is important to notice, however, that in the aforementioned spoken languages, the morphology used for active constructions is not the same for passive, reflexive, and reciprocal constructions. Another question is whether personal pronouns in lieu of reflexive pronouns can be used for reflexive expressions in ASL as in the case of PRO.1 CONVINCE.1, but this has not yet been investigated (Erin Wilkinson, personal communication).

11 This sign. FLATTER can be found at: https://aslsignbank.haskins.yale.edu/dictionary/gloss/3409.html

12 This sign FOOL^14^ can be found at: https://aslsignbank.haskins.yale.edu/dictionary/gloss/1491.html.

13 Hou (2022) found that there is a statistical preference for PRO.1 occurring in the s-1 slot to collocate with LOOK/“reaction” over LOOK/“vision,” SEE and SEE-SEE.

14
https://www.youtube.com/watch?v=zlR2EGi6_wA
