# Supplementary material for: A Usage-Based Proposal for Argument Structure of Directional Verbs in American Sign Language
Source: Front Psychol. 2022 May 17;13:808493. doi: 10.3389/fpsyg.2022.808493 (PMC9157181; doi:10.3389/fpsyg.2022.808493)
Supplement: Supplementary file 1 [file Data_Sheet_1.pdf]

Supplementary Material

Links to videos used for Dataset 1

ASLized! (2017). ASL in Academic Settings: Language Features. Link: https://www.youtube.com/watch?v=VX18-4m-EN0

ASLonline. (2015). ASLonline3 Unit 21 – Feature Movies: Babysitting Blunder. Link: https://www.youtube.com/watch?v=pAKTzMwvG9c

ASLonline. (2015). ASLonline3 Unit 25 – Feature Movies: Cultural Anecdote: Working out at the Gym. Link: https://www.youtube.com/watch?v=_uOpXGJYUcc

Frye, Callie. (2020). DCARA March 20, 2019. Link: https://www.youtube.com/watch?v=kVlVQaTr7Mk&feature=youtu.be

The Daily Moth. (2017). The Daily Moth 2-27-17. Link: https://www.youtube.com/watch?v=CI-zaV-6WDA

VirtualDeafChurch. (2014). I have become an Atheist. Link: https://www.youtube.com/watch?v=x1q3GiTJzfM&feature=youtu.be

Street Leverage. (2012). Trudy Suggs: Deaf Disempowerment and Today's Interpreter. Link: https://www.youtube.com/watch?v=pDSNKRaOmo8

Links to videos used for Dataset 2

Note: Some of the videos are on YouTube or Vimeo. Two are in a public group on Facebook. Other videos are not listed here; they are either no longer available for public viewing or missing.

ASLized! (2017). ASL in Academic Settings: Language Features. Link: https://www.youtube.com/watch?v=VX18-4m-EN0

ASLized! (2013). Deaf Schools (with audio and captions). Link: https://www.youtube.com/watch?v=mkwYHheJQVw

ASLonline. (2015). ASLonline Unit 20 – Feature Movies: Games: Poker. Link: https://www.youtube.com/watch?v=NgethUZ2hCM

ASLonline. (2015). ASLonline Unit 20 – Feature Movies: World Traveling and Cultures. Link: https://www.youtube.com/watch?v=I8mvq9xZHTc

ASLonline. (2015). ASLonline3 Unit 21 – Feature Movies: Babysitting Blunder. Link: https://www.youtube.com/watch?v=pAKTzMwvG9c

ASLonline (2015). ASLonline3 Unit 23 – Feature Movies: Career Experiences. Link: https://www.youtube.com/watch?v=vjdPoxzI2Z4

ASLonline. (2015). ASLonline3 Unit 23 – Feature Movies: Renny's ASL Class. Link: https://www.youtube.com/watch?v=NGCYhSATsVw

ASLonline. (2015). ASLonline3 Unit 25 – Feature Movies: Cultural Anecdote: Working out at the Gym. Link: https://www.youtube.com/watch?v=_uOpXGJYUcc

ASLonline. (2015). ASLonline3 Unit 25 – Feature Movies: Froggie's Story. Link: https://www.youtube.com/watch?v=Juuh9JXE-5c&feature=youtu.be

ASLonline. (2015). ASLonline3 Unit 25 – Feature Movies: Mafia Joke. Link: https://www.youtube.com/watch?v=bsaJLywU7b0

ASL THAT! (2017). Signing Naturally Numbers 6-9 Double tap? Link: https://www.facebook.com/groups/ASLTHAT/permalink/2024014911163339/

Baer, Joey. (2017). ASLTA or ASLRT? Link: https://www.youtube.com/watch?v=TGcNCkYmUWU

Baer, Joey. (2017). ASLTA or ASLRT Follow up Vlog. Link: https://www.youtube.com/watch?v=yzR_9n7xJyM

Baer, Joey. (2015). What about Academic ASL? Link: https://www.youtube.com/watch?v=iySP84byPk4

Baer, Joey. (2015). Cyberbullying & Community Accountability. Link: https://www.youtube.com/watch?v=UOYbz2uVzUM&feature=youtu.be

Baer, Joey. (2013). ASDB #1 vlog: Need your help. Link: https://www.youtube.com/watch?v=23k5A7k-GZ4&feature=youtu.be

Baer, Joey. (2013). ASDB Vlog #2. Link: https://www.youtube.com/watch?v=juMljMsgrO0

Baer, Joey. (2013). ASDB Vlog #4: We must hold ENTIRE board accountable. Link: https://www.youtube.com/watch?v=9U6gaEM6DpE

Baer, Joey. (2013). ASBD #6 Vlog: Fight Continues for Social Justice. Link: https://www.youtube.com/watch?v=VJjUOZorVHk

Baer, Joey. (2013). Stop sim-com! Or am I mistaken? Link: https://www.youtube.com/watch?v=qr2lFaJEh_I&feature=emb_logo

Bienvenu, Martina. (2018). Purification of ASL, no! Link: https://www.youtube.com/watch?v=2zXhE5gv3Pw&t=5s

Frye, Callie. (2020). DCARA March 20, 2019. Link: https://www.youtube.com/watch?v=kVlVQaTr7Mk&feature=youtu.be

McFeely, Sheena. (2011). The Pearls – Leah Katz Hernandez/ Link: https://www.youtube.com/watch?v=zlR2EGi6_wA

Pink Dippers. (2015). Deafdar? Link: https://www.facebook.com/PinkDippers/videos/196743384001711/

Savage, Jon Lenois. (2007). Interview with Protestor & arrested on "Black Friday" (ASL). Link: https://www.youtube.com/watch?v=UOIuOxl9_XQ

Street Leverage. (2012). Trudy Suggs: Deaf Disempowerment and Today's Interpreter. Link: https://www.youtube.com/watch?v=pDSNKRaOmo8

The Daily Moth. (2016). The Daily Moth 4-12-16. Link: https://www.youtube.com/watch?v=-XfqzlNNrgU

The Daily Moth. (2016). The Daily Moth 12-12-16. Link: https://www.youtube.com/watch?v=kJowhXtFr2w

The Daily Moth. (2016). The Daily Moth 12-14-16. Link: https://www.youtube.com/watch?v=ogzmaePy6gg (00:02:01-00:05:10 only)

The Daily Moth. (2016). The Daily Moth 12-20-16. Link: https://www.youtube.com/watch?v=AGFFRsBOIxQ (00:02:28-00:06:50 only)

The Daily Moth. (2017). The Daily Moth 1-2-17. Link: https://www.youtube.com/watch?v=JqQT2rcWT-s (00:03:28-00:00:04:55 only)

The Daily Moth. (2017). The Daily Moth 1-17-16. Link: https://www.youtube.com/watch?v=z0_XPr3ZKhk (00:10:02-00:16:43 only)

The Daily Moth. (2017). The Daily Moth 1-23-17. Link: https://www.youtube.com/watch?v=sgSqwCOoZms (00:08:29-00:11:46 only)

The Daily Moth. (2017). The Daily Moth 2-2-17. Link: https://www.youtube.com/watch?v=LBBHVvHM4As

The Daily Moth. (2017). The Daily Moth 2-13-17. Link: https://www.youtube.com/watch?v=Ge8tepq-9bQ

The Daily Moth. (2017). The Daily Moth 2-20-17. Link: https://www.youtube.com/watch?v=3NkoX0RZKgE

The Daily Moth. (2017). The Daily Moth. 2-22-17. Link: https://www.youtube.com/watch?v=ohrr3PHkEQE

The Daily Moth. (2017). The Daily Moth. 5-8-17. Link: https://www.youtube.com/watch?v=kefejHhNbH8

The Daily Moth. (2017). The Daily Moth. 5-10-17. Link: https://www.youtube.com/watch?v=Dini1GDT-qo

The Daily Moth. (2017). The Daily Moth. 6-1-17. Link: https://www.youtube.com/watch?v=VtK-FGONft4

The Daily Moth. (2017). The Daily Moth. 8-11-17. Link: https://www.youtube.com/watch?v=1AfNo1OJoOs&t=84s

The Daily Moth. (2017). The Daily Moth. 8-26-17. Link: https://www.facebook.com/watch/?v=738826366319266

The Daily Moth. (2018). Early Intervention for Deaf POC children. Link: https://www.youtube.com/watch?v=wlhdq52cDLI&feature=youtu.be

The Daily Moth. (2019). The Daily Moth 3-22-2019. Link: https://www.youtube.com/watch?v=rTJt6dTwc0k&feature=youtu.be (00:14:32-00:20:15 only)

The Daily Moth. (2020). COVID-19 disproportionately impacting Black and Latinx populations. Link: https://www.youtube.com/watch?v=hvsIMRUhF3g

The Daily Moth. (2020). Q & A with Deaf Gamer: DM3. Link: https://www.youtube.com/watch?v=969xm4nuwv4

Timm, Rosa Lee. (2016). A Deaf Take on Music. Link: http://hdl.handle.net/2047/D20197917

T.S. Writing Services. (2016). Awkward! Salary requests. Link: https://vimeo.com/155917950

T.S. Writing Services. (2016). Tips for writing image descriptions. Link: https://vimeo.com/159867956

T.S. Writing Services. (2016). Myth: Good Writer = Good Editor. Link: https://vimeo.com/161654942

T.S. Writing Services. (2016). Four myths about plagiarism. Link: https://vimeo.com/162773246

T.S. Writing Services. (2016). Fact or Fiction? Three Steps to Take. Link: https://vimeo.com/164427880

T.S. Writing Services. (2017). The dangers of not checking facts. Link: https://vimeo.com/217773097

T.S. Writing Services. (2017). Responding to negative reviews online. Link: https://vimeo.com/228007346

T.S. Writing Services. (2017). How posts can affect people's perceptions of you. Link: https://vimeo.com/241925863

T.S. Writing Services. (2017). Including images in social media posts. Link: https://vimeo.com/242984769

T.S. Writing Services. (2017). Social media: To share or not to share. Link: https://vimeo.com/243973891

T.S. Writing Services. (2018). Supporting Deaf Authors: About Savory Words Publishing. Link: https://vimeo.com/252449291

T.S. Writing Services. (2018). The first English and ASL dictionaries. Link: https://vimeo.com/254679442

T.S. Writing Services. (2018). How are dictionary entries chosen? Link: https://vimeo.com/255666898

T.S. Writing Services. (2018). Unique dictionaries within the Deaf community. Link: https://vimeo.com/256707462

VirtualDeafChurch. (2014). I have become an Atheist. Link: https://www.youtube.com/watch?v=x1q3GiTJzfM&feature=youtu.be
